# Assessment of Non-Cereal Products Gluten Cross-Contamination Exposure Risk in a Polish Female Population of Patients Diagnosed with Coeliac Disease

**DOI:** 10.3390/nu17071281

**Published:** 2025-04-06

**Authors:** Dominika Skolmowska, Dominika Głąbska, Dominika Guzek, Frank Vriesekoop

**Affiliations:** 1 Department of Dietetics, Institute of Human Nutrition Sciences, Warsaw University of Life Sciences (SGGW-WULS), 159C Nowoursynowska Street, 02-776 Warsaw, Poland; dominika_glabska@sggw.edu.pl; 2 Department of Food Market and Consumer Research, Institute of Human Nutrition Sciences, Warsaw University of Life Sciences (SGGW-WULS), 159C Nowoursynowska Street, 02-776 Warsaw, Poland; dominika_guzek@sggw.edu.pl; 3 Harper Food Innovation, Harper Adams University, Newport TF10 8NB, UK; fvriesekoop@harper-adams.ac.uk

**Keywords:** coeliac disease, gluten, cross-contamination, exposure, gluten-free products, availability, consumers

## Abstract

Background/Objectives: Following gluten-free diet is challenging, due to risk of gluten cross-contamination. The study aimed to assess the non-cereal products gluten cross-contamination exposure risk in patients with coeliac disease. Methods: The study was conducted in a population of 699 Polish female members of the Polish Coeliac Society purchasing gluten-free products on-line (445 patients, 254 relatives). Participants were asked about frequency of buying and availability of gluten-free alternatives of non-cereal products characterized by the gluten cross-contamination risk (‘hidden’ gluten sources). Results: The most frequently bought non-cereal gluten-free alternatives of the ‘hidden’ gluten sources were baking powders, spices, side dishes, ice cream, chocolate and chocolate products, snack bars and candies. The caregivers often declared buying ‘often’ gluten-free baking powder, snack bars, chocolate and chocolate products, candies, ice cream, as well as often declared problems with the availability of gluten-free spices, chocolate and chocolate products, while patients often declared buying ‘often’ gluten-free beer, as well as often declared problems with its availability. The older respondents often declared buying ‘often’ gluten-free baking powder, while younger respondents often declared buying ‘often’ gluten-free chocolate and chocolate products, as well as often declared problems with the availability of gluten-free instant soups, and beer. The respondents living in small towns/villages often declared problems with the availability of gluten-free powder sauces. The respondents not purchasing in hypermarkets often declared buying ‘often’ gluten-free baking powder, spices, candies. The respondents who most often purchased gluten-free products often declared problems with the availability of gluten-free side dishes, chocolate and chocolate products. Conclusions: The majority of patients diagnosed with coeliac disease do not buy a number of gluten-free alternatives of the ‘hidden’ gluten sources, so they may be prone to gluten exposure, due to non-cereal products’ gluten cross-contamination risk.

## 1. Introduction

Coeliac disease, also known as ‘coeliac sprue’ or ‘gluten-sensitive enteropathy’, is a chronic inflammatory disease of the small intestine with an inappropriate immune-mediated reaction that is triggered by gluten ingestion and manifested in genetically susceptible individuals [1,2]. Coeliac disease is one of the most common chronic disorders [3]. According to the systematic review and meta-analysis of Singh et al. [4], it is estimated that the global prevalence of coeliac disease based on the results of serologic tests is 1.4%, while based on biopsy results is 0.7%. The prevalence of coeliac disease differs depending on sex, and region of the world, as it is more frequently diagnosed in women than men, as well as in Europe and Oceania than in the other regions [4]. Clinical presentation of patients with coeliac disease is various, ranging from classical and non-classical gastrointestinal symptoms, or extraintestinal manifestations to sub-clinical cases [5].

Currently, the recommended and effective treatment for coeliac disease is strict adherence to a lifelong gluten-free diet [6], and it is advised to do it under the supervision of a healthcare providers, including a dietitian [7]. It is associated with the elimination of gluten, being a heterogeneous group of storage proteins which are found in various cereal grains, namely wheat, rye, barley, hybrids (for example, emmer or triticale), and their products, such as malt [8]. A gluten-free diet means the complete exclusion from the diet of both these products and the products that may be cross-contaminated with gluten to limit the total daily gluten intake, while the level to be obtained is defined by the recommendations of the British Society of Gastroenterology, as below 10 mg a day [9]. Such a reduction of daily gluten intake is associated with several beneficial health outcomes for coeliac disease patients, including the healing of the duodenal mucosa, resulting in the alleviation of disease symptoms [6], prevention of coeliac disease complications [10], and the improvement of the patients’ quality of life [11].

In order to allow following a gluten-free diet, the category of gluten-free products was established and defined as containing less than 20 mg of gluten/1 kg of food product (20 ppm of gluten) [12]. However, processed gluten-free products demonstrate higher amounts of fats, trans fatty acids, and salt than gluten-containing counterparts [13]. Moreover, it is stated that gluten-free products often contain lower levels of protein, iron, folic acid, thiamin, riboflavin, niacin, and fiber compared to products naturally containing gluten, so as a result, a gluten-free diet may have improper nutritional quality [14].

It is indicated that adhering to a gluten-free diet is very challenging for coeliac patients due to the ubiquitous gluten prevalence not only in food products but also in medicines, as it is frequently used as an additive [15]. However, even if they choose gluten-free alternatives to products obtained from gluten-containing cereals, the other possible sources of gluten are non-certified products, made of the other cereals, as they are often processed on the same production line as gluten-containing grains, which may result in gluten contamination [16]. Another problematic issue for coeliac patients is that they may even know the obvious cereal products that should be replaced with gluten-free alternatives; however, they may not be aware of such necessity in terms of some products like sauces, canned soups, broth, or beer, which may be a gluten source as well [17]. It is estimated that despite the efforts of coeliac patients to pay attention to the proper strict gluten-free diet, intentional gluten consumption occurs in 40% of coeliac individuals, while the frequency of unintentional gluten consumption may be even higher [18].

Another challenge for coeliac patients may be a ‘hidden’ gluten, as even patients characterized by a strong adherence to a gluten-free diet may unknowingly consume it in a hidden way [19]. ‘Hidden’ gluten may be presented in naturally gluten-free products, or non-cereal products, or cereal ones produced from non-gluten cereals, including a number of non-certified processed foods, medications, and cosmetics, due to cross-contamination, or applied additives [16]. So, the ‘hidden’ gluten sources may be defined as products that may contain gluten but are not perceived in an obvious way as they do so, or the gluten content is not mentioned on a product label in a way that is easy to understand.

Poland in the international comparison, based on the systematic review and meta-analysis by Singh et al. [4], was stated to be characterized by a relatively low coeliac disease prevalence, being in the first quartile for the seroprevalence rate. It corresponds with the commonly indicated for Poland low availability of gluten-free products [20], and a common for Polish products lack of information on non-cereal products labels as to whether they are ‘gluten-free’, so the risk of cross-contamination in the production plant may occur depending on the batch [21]. As a result, in the recent Polish study by Kostecka et al. [22], it was indicated that for Polish coeliac disease patients the main problem is the risk of the accidental gluten exposure especially in products that were not labeled as ‘gluten-free’, which may be supposed to appear mainly for ‘hidden’ gluten sources. Considering the described situation, it was hypothesized that due to low availability of gluten-free alternatives of the ‘hidden’ gluten sources, there is a gluten cross-contamination exposure risk in a Polish female population of patients diagnosed with coeliac disease.

Taking into account the obstacles mentioned above associated with following a gluten-free diet, the presented study aimed to assess the non-cereal products gluten cross-contamination exposure risk in a Polish female population of patients diagnosed with coeliac disease, based on the frequency of buying and availability of gluten-free alternatives of the ‘hidden’ gluten sources declared in a population of patients and their caregivers. The study was planned to be conducted in a population of women only, as in Poland, similarly as in the other countries [23], woman are in families responsible for meals planning, foods purchasing and meals preparation [24]. The other arguments for assessing only population of women are the fact that women are stated to be more interested in health than men [25], and for women there is a higher frequency of coeliac disease than for men [26]. Taking this into account, the equal approach was chosen in the other Polish studies of the gluten-free products choices focusing on female patients only [27].

## 2. Materials and Methods

### 2.1. General Information

The study was conducted within a project developed to characterize the situation of coeliac disease patients from an international perspective while assessing in various countries their patients’ beliefs and opinions about gluten-free products, as well as the availability and prices of gluten-free products [20,28]. The presented part of the project was conducted within partnership of the Institute of Human Nutrition Sciences, Warsaw University of Life Sciences (SGGW-WULS) in Poland (in charge of data gathering and analysis), Polish Coeliac Society (in charge of questionnaire dissemination within Society members), and Harper Adams University in the United Kingdom (in charge of project administration and coordination of actions). All the actions associated with this part of the project were carried out in the period of May–August 2022.

All study participants provided their informed consent to participate in the study. The study was conducted based on the Declaration of Helsinki and within the ethical approval of Harper Adams University, United Kingdom (No. 0439-202106-STAFF-CO2).

### 2.2. Participants of the Study

The gathered study sample comprised of patients diagnosed with coeliac disease and their caregivers, being the members of the Polish Coeliac Society and recruited to the study by the Society. Polish Coeliac Society is a member of the Association of European Coeliac Societies (AOECS) and is engaged in scientific research covering coeliac disease issues in a population of Polish patients [29].

The inclusion criteria were determined as follows:

(1) Membership of the Polish Coeliac Society.
(2) Being a patient diagnosed with coeliac disease or their caregiver.
(3) Acknowledging the informed consent before the study.

The exclusion criteria were determined as follows:

(1) Male gender.
(2) Age of below 18.
(3) Declared not living in Poland.
(4) Declared not purchasing gluten-free products.
(5) Declared not purchasing gluten-free products online even occasionally.

The initial sample size was 978 participants who completed the questionnaire; however, 159 participants were excluded from the analysis based on the exclusion criteria (of whom 95 participants were males, 36 participants were <18 years old, 2 participants declared not living in Poland, 26 participants declared not purchasing gluten-free products, and 120 participants declared not purchasing gluten-free products on-line at least occasionally). Therefore, the gathered final sample size was 699 participants.

Since the study intended to assess the non-cereal products’ gluten cross-contamination risk, based on the frequency of buying and availability of gluten-free alternatives of the ‘hidden’ gluten sources, it must have been taken into account that some products from this group are not so easily available. Considering this, the study was planned to be conducted in a population of coeliac patients declaring purchasing gluten-free products online at least occasionally to define the population as having theoretically similar access to all the products available on the Polish market.

### 2.3. Applied Questionnaire and Data Analysis

The questionnaire applied for the study was a universal tool developed to be used independently of the studied country to characterize the situation of coeliac disease patients from an international perspective while assessing in various countries their patients’ beliefs and opinions about gluten-free products, as well as the availability and prices of gluten-free products. The questionnaire and its structure were characterised in detail in the previous study [28], while the adaptation of the questionnaire for a Polish population was described in the other study conducted in Poland [20]. While the study covered a broader subject area, the presented analysis focuses on the frequency of buying gluten-free alternatives of the ‘hidden’ gluten sources and problems with their availability.

The study was conducted using the computer-assisted web interview (CAWI) method, while the questionnaire was distributed to a population of patients diagnosed with coeliac disease and their caregivers, being the members of the Polish Coeliac Society and no snowball effect was allowed.

Within the conducted study, the respondents were asked about the frequency of buying gluten-free alternatives of non-cereal products characterized by the gluten cross-contamination risk, namely so-called ‘hidden’ gluten sources, as well as about problems with the availability. The list of such products was based on the materials by the Celiac Disease Foundation [30] but adapted for the Polish market products, as follows: baking powder, snack bars, powder sauces, soups, stock cubes, side dishes, spices, instant soups, chocolate and chocolate products, candies, ice cream, and beer. The listed products included the most commonly consumed gluten-free alternatives of non-cereal products among Polish coeliac patients. Additionally, two questions about non-food products were added to verify participants’ approach for the other groups of products, and the questions about body and face cosmetics, as well as hair care cosmetics, were included.

For each group of products, the participants were asked about how often they purchase gluten-free alternatives (labelled and distributed as gluten-free ones), while they were allowed to describe their declared frequency while using one of the 4 categories: never, rarely, sometimes, often. Afterwards, for each group of gluten-free alternatives of non-cereal products, participants who declared purchasing it at least rarely (after excluding those responding ‘never’ for the question about the frequency of buying) were asked about problems with the availability of gluten-free alternatives. For each group of products, the participants were asked if they have an experience associated with gluten-free alternatives missing in the stores, while they were allowed to indicate that either they have or do not have problems with the availability.

Additionally, participants were asked some questions about their basic characteristics to assess determinants influencing the non-cereal products gluten cross-contamination exposure risk in a Polish female population of patients diagnosed with coeliac disease. The following determinants were assessed:

− Diagnosis of coeliac disease (assessed based on single-choice closed-ended question with options of: being diagnosed with coeliac disease and having a relative diagnosed with coeliac disease to choose from)—participants stratified into following subgroups: (1) diagnosed with coeliac disease, (2) caregiver of a patient with coeliac disease.
− Age (assessed based on single-choice closed-ended question with options of: <18 years, 18–24 years, 25–34 years, 35–44 years, 45–54 years, 55–64 years, 65–74 years, 75–84 years, and >84 years)—participants stratified into following subgroups: (1) age < 35 years, (2) age ≥ 35 years (after aggregation of age categories into categories of (1) young adults and (2) middle-aged and older adults, according to the categorization applied by the other authors [31]).
− Place of residence (assessed based on open-ended question about the zip code)—participants stratified into following subgroups: (1) living in a big city, (2) living in a small town/village (after interpretation based on the number of inhabitants for a cutoff of 100,000 inhabitants, on the basis of the categorization by the Central Statistical Office in Poland for VI class and VII class of the size of cities [32] and according to the categorization applied by the other authors [33]).
− Primary place of purchasing major grocery shopping (assessed based on open-ended question about the names of the stores/markets)—participants stratified into following subgroups: (1) purchasing in hypermarkets, (2) purchasing in shops other than hypermarkets (after interpretation based on the size of the store/market for a cutoff of 2500 m^2^, on the basis of the categorization by the Central Statistical Office in Poland [34] and according to the trade press retail market analysis [35]).

Last but not least, within the general characteristics, the participants were asked if they declare (1) a general problem with the availability of gluten-free products and (2) a general problem with the quality of gluten-free products, while they were allowed to indicate that either they have or do not have problems with the availability/quality of gluten-free products.

The questionnaire used for the study was developed within an international project to characterize the situation of coeliac disease patients in various countries, so the same tool was to be used in all participating countries. The original version of the questionnaire was developed in English, and afterwards it was adapted for other countries, while translation into national language and transcultural adaptation were applied. As recommended by the World Health Organization (WHO) [36] and according to the commonly applied procedure [37], the process included translation from English to Polish (forward translation), translation from Polish to English (backward translation), and final tool polishing to guarantee the equivalence of the obtained tool on a conceptual, semantic, idiomatic, and cultural level. More information about questionnaire adaptation for Polish version was presented in previous publication [20].

In order to reduce the risk of bias during the study, for the selection bias and the observation bias specific actions were planned. For the selection bias, to avoid under-representation, or over-representation within the sub-groups, the recruitment was conducted in cooperation with the Polish Coeliac Society, as the members of the Society represent the coeliac disease patients and their caregivers from all the regions of Poland (to increase the chance of gathering the sample representative for various regions). For the observation bias, to avoid situation when respondents aware of the fact of being observed, change their behaviors (declare different behaviors), the questionnaire was introduced and explained as associated with Polish market of gluten-free products (to not cause the impression, that respondents are observed and assessed, but that their answers are necessary to assess Polish market of gluten-free products).

### 2.4. Statistical Analysis

Descriptive statistics were used to summarize the categorical data. The results are presented as frequencies and percentages. The frequency of buying and problems with the availability of gluten-free alternatives of the ‘hidden’ gluten sources declared in the studied Polish female population of patients diagnosed with coeliac disease and their caregivers were compared independently in the sub-groups, stratified by diagnosis of coeliac disease, age, place of residence, and primary place of purchasing major grocery shopping.

The statistical analysis was based on the chi^2^ test applied to compare the frequency in sub-groups. The *p* ≤ 0.05 was interpreted as a statistically significant level. The effect size was presented as Cramer’s V (for non 2 × 2 contingency tables) and phi (φ) (for a 2 × 2 contingency tables). The statistical analysis was conducted using Statistica 13.0 (Statsoft Inc., Tulsa, OK, USA).

## 3. Results

The general characteristics of the studied Polish female population of patients diagnosed with coeliac disease and their caregivers declaring at least occasionally purchasing gluten-free products online, is presented in Table 1. The majority of respondents were aged 35–44 (46%), inhabited small towns or villages (53%), indicated hypermarkets as the major place of grocery shopping (71%), were diagnosed with coeliac disease (64%), and declared problem with gluten-free products availability (97%), and problem with gluten-free products quality (62%).

The frequency of buying gluten-free alternatives of the ‘hidden’ gluten sources declared in the studied Polish female population of patients diagnosed with coeliac disease and their caregivers declaring at least occasionally purchasing gluten-free products online, is presented in Table 2. The most frequently bought non-cereal gluten-free alternatives of the ‘hidden’ gluten sources were baking powders and spices, as 46% of the respondents declared that they often purchase baking powders, and the same share declared it for spices. Less frequently were purchased side dishes (38% of respondents declaring that they buy it often), ice cream (33%), chocolate and chocolate products (25%), snack bars (18%), and candies (17%). However, while combining respondents declaring purchasing listed products often and sometimes, for each of them, a vast majority of respondents bought, in the following order: baking powder (81%), side dishes (77%), ice cream (74%), spices (72%), chocolate and chocolate products (72%), snack bars (56%), candies (53%). On the other hand, some gluten-free products were indicated by respondents as hardly purchased, such as instant soups (84% of respondents declaring that they never buy them), powder sauces (80%), soups (73%), and beer (56%). A little bit more frequently were purchased stock cubes (66% of respondents declaring that they buy them never or rarely), while for non-food products, the situation was similar (hair care cosmetics—57%, body and face cosmetics—55.5%).

The problems with the availability of gluten-free alternatives of the ‘hidden’ gluten sources declared in the studied Polish female population of patients diagnosed with coeliac disease and their caregivers declaring at least occasionally purchasing gluten-free products online, are presented in Table 3. In a sub-group of individuals purchasing listed gluten-free products, the problems with their availability were declared mainly for beer (43%), instant soups (36%), soups (36%), spices (32%), and baking powder (29%).

The frequency of buying gluten-free alternatives of the ‘hidden’ gluten sources declared in the studied Polish female population of patients diagnosed with coeliac disease and their caregivers declaring at least occasionally purchasing gluten-free products online, stratified by diagnosis of coeliac disease, is presented in Table 4. It was stated that caregivers of coeliac disease patients more often declared buying ‘often’ gluten-free products, such as baking powder (*p* = 0.0005), snack bars (*p* = 0.0106), chocolate and chocolate products (*p* = 0.0401), candies (*p* < 0.0001), and ice cream (*p* = 0.0003) compared to respondents diagnosed with coeliac disease. On the other hand, coeliac patients more often declared buying ‘often’ gluten-free beer (*p* < 0.0001) compared to the caregivers.

The frequency of buying gluten-free alternatives of the ‘hidden’ gluten sources declared in the studied Polish female population of patients diagnosed with coeliac disease and their caregivers declaring at least occasionally purchasing gluten-free products online, stratified by age, is presented in Table 5. It was stated that older respondents more often declared buying ‘often’ gluten-free products, such as baking powder (*p* = 0.0392), compared to younger ones. On the other hand, younger respondents more often declared buying ‘often’ gluten-free chocolate and chocolate products (*p* = 0.0307), as well as body and face cosmetics (*p* = 0.0294) compared to older ones.

The frequency of buying gluten-free alternatives of the ‘hidden’ gluten sources declared in the studied Polish female population of patients diagnosed with coeliac disease and their caregivers declaring at least occasionally purchasing gluten-free products online, stratified by place of residence, is presented in Table 6. There were no statistically significant differences between respondents living in big cities and those living in small towns or villages in terms of buying the studied products.

The frequency of buying gluten-free alternatives of the ‘hidden’ gluten sources declared in the studied Polish female population of patients diagnosed with coeliac disease and their caregivers declaring at least occasionally purchasing gluten-free products online, stratified by place of purchasing major grocery shopping, is presented in Table 7. It was stated that respondents purchasing in shops other than hypermarkets more often declared buying ‘often’ gluten-free products, such as baking powder (*p* = 0.0004), spices (*p* = 0.0369), and candies (*p* = 0.0371) compared to those purchasing in hypermarkets.

The problems with the availability of gluten-free alternatives of the ‘hidden’ gluten sources declared in the studied Polish female population of patients diagnosed with coeliac disease and their caregivers declaring at least occasionally purchasing gluten-free products online, stratified by diagnosis of coeliac disease, are presented in Table 8. It was stated that caregivers of coeliac patients more often than patients diagnosed with coeliac disease declared problems with the availability of gluten-free products, such as spices (*p* < 0.0001), as well as chocolate and chocolate products (*p* = 0.0317). On the other hand, coeliac patients more often than caregivers of coeliac patients declared problems with the availability of gluten-free beer (*p* < 0.0001).

The problems with the availability of gluten-free alternatives of the ‘hidden’ gluten sources declared in the studied Polish female population of patients diagnosed with coeliac disease and their caregivers declaring at least occasionally purchasing gluten-free products online, stratified by age, are presented in Table 9. It was stated that younger respondents more often declared problems with the availability of gluten-free products, such as instant soups (*p* = 0.0415), and beer (*p* = 0.0015), compared to older ones.

The problems with the availability of gluten-free alternatives of the ‘hidden’ gluten sources declared in the studied Polish female population of patients diagnosed with coeliac disease and their caregivers declaring at least occasionally purchasing gluten-free products online, stratified by place of residence, are presented in Table 10. It was stated that respondents living in small towns or villages more often declared problems with the availability of gluten-free products, such as powder sauces (*p* = 0.0094), body and face cosmetics (*p* = 0.0102), as well as hair care cosmetics (*p* = 0.0186), compared to those living in big cities.

The problems with the availability of gluten-free alternatives of the ‘hidden’ gluten sources declared in the studied Polish female population of patients diagnosed with coeliac disease and their caregivers declaring at least occasionally purchasing gluten-free products online, stratified by primary place of purchasing major grocery shopping, are presented in Table 11. There were no statistically significant differences between respondents purchasing in hypermarkets and purchasing in shops other than hypermarkets in terms of problems with the studied products.

**Figure 1:**
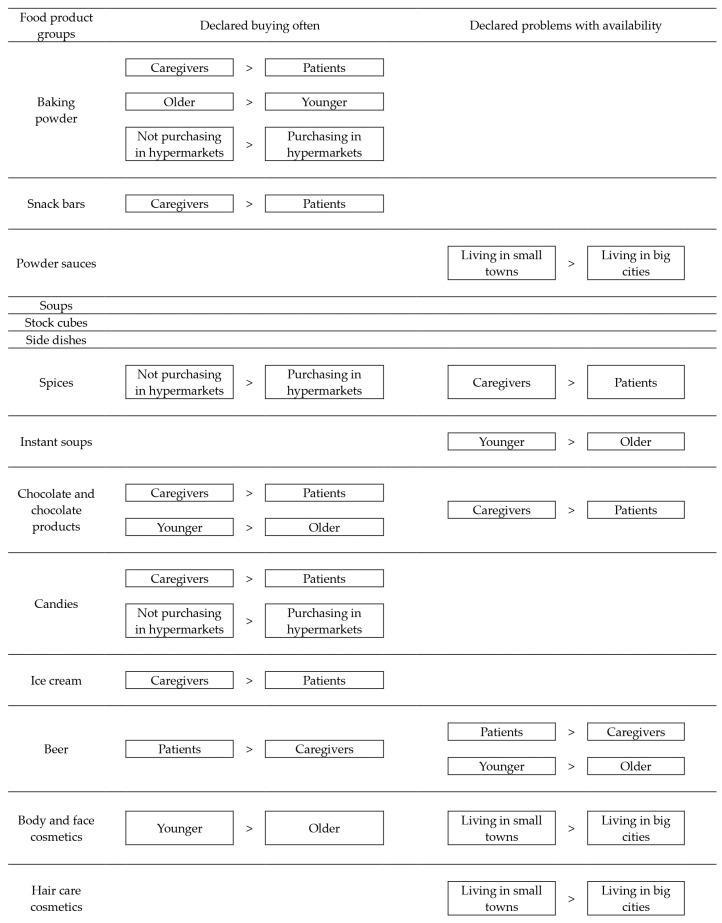
The graphical summary of the results of the frequency of buying and problems with the availability of gluten-free alternatives of the ‘hidden’ gluten sources declared in the studied Polish female population of patients diagnosed with coeliac disease and their caregivers declaring at least occasionally purchasing gluten-free products online.

The graphical summary of the results of the frequency of buying and problems with the availability of gluten-free alternatives of the ‘hidden’ gluten sources declared in the studied Polish female population of patients diagnosed with coeliac disease and their caregivers declaring at least occasionally purchasing gluten-free products online is presented in Figure 1. It was observed that the agreement of the observations were stated for chocolate and chocolate products, as well as for beer, as for caregivers of coeliac disease patients were stated both higher frequency of buying and higher problems with availability of chocolate and chocolate products than for coeliac disease patients, while for coeliac disease patients were stated both higher frequency of buying and higher problems with availability of beer than for caregivers of coeliac disease.

The problems with the availability of gluten-free alternatives of the ‘hidden’ gluten sources declared in the studied Polish female population of patients diagnosed with coeliac disease and their caregivers declaring at least occasionally purchasing gluten-free products online, stratified by the frequency of buying them, are presented in Table 12. It was stated that respondents who most often purchased gluten-free products, while compared with those who purchased them less often, more often declared problems with the availability of gluten-free products, such as side dishes (*p* < 0.0001), chocolate and chocolate products (*p* = 0.0146), body and face cosmetics (*p* = 0.0350), as well as hair care cosmetics (*p* = 0.0284).

## 4. Discussion

Within the present study, it was shown that the most frequently bought non-cereal gluten-free alternatives of the ‘hidden’ gluten sources were baking powders and spices, side dishes, ice cream, chocolate and chocolate products, snack bars, and candies. However, such choices may vary depending on the study. In the study of Dean et al. [38], it was noted that in a group of individuals with coeliac disease, apart from gluten-free cereal products, the most frequently purchased gluten-free products were chocolate and ice cream. Choosing gluten-free alternatives or the typical non-cereal products prone to gluten cross-contamination exposure risk depends on many factors, as well as it may either cause changes in intestinal mucosa and symptoms of the disease or not.

While mentioning the role of the observed symptoms, the study by Zingone et al. [39] should be discussed, which described a case of a man, being food blogger and sommelier, as well as described as a connoisseur of beer, who was monitored during 4 weeks of gluten exposure in daily beer consumption (up to 4 L per day, with the gluten content of 150–800 mg/L) and in this period neither any relevant symptom nor the pathological level of serum antibodies was stated, which was explained by an individual sensitivity. Similar observations were formulated in the study assessing a group of coeliac disease patients declaring voluntary and occasional gluten exposure in products not being gluten-free, while the groups of cereal products, sweets, and beer were studied, and it was concluded that such gluten exposure did not influence any significant clinical symptom or small bowel damage, suggesting reaching some degree of gluten tolerance, which should always be assessed individually [40].

However, it should be supposed that the majority of patients who do not perfectly adhere to a gluten-free diet and consume some gluten-containing products, including non-cereal products, do not do it believing in their individual sensitivity and developing gluten tolerance, but they probably do not realize the risk of gluten cross-contamination exposure. It may be confirmed by the results of the study defining major reasons for poor diet adherence in coeliac patients, listed as age at diagnosis (higher adherence in patients diagnosed later in life), coexisting depression, high cost of gluten-free products (both negatively influencing adherence), symptoms on ingestion of gluten, nutrition counselling, knowledge of gluten-free products, understanding of food labels, availability of gluten-free products, receiving gluten-free products on prescription and membership of a coeliac society (all of them positively influencing adherence) [41]. Similar observations were formulated in the other study, as the higher adherence in coeliac patients was promoted by: understanding of the gluten-free diet, membership of a celiac disease advocacy group, and perceived ability to maintain adherence despite travel or changes in mood or stress [42].

What should be emphasized based on the referred studies, is the role of knowledge on the one side (understanding of gluten-free diet, nutrition counselling, membership in dedicated societies, belief in the gluten-free diet), and availability on the other side (prices of gluten-free products, availability in shops, receiving gluten-free products on prescription). The role of knowledge of coeliac patients was confirmed in the study of Vázquez-Polo et al. [43], as some products were indicated as causing suspicion in patients with coeliac disease, including processed food with a long list of ingredients, which probably results from lack of understanding of food label information.

The present study found that there may be problems with the availability of some gluten-free non-cereal products, mainly beer, instant soups, soups, spices, and baking powder, even if they are at least occasionally purchased online. This finding is consistent with those from other studies, indicating that it may be troublesome for coeliac patients to buy gluten-free alternatives to gluten-containing products. A study by Dean et al. [38] analyzing the data from 13 European countries revealed that 79% of patients with coeliac disease, patients with gluten intolerance, or their caregivers experienced problems with the availability of gluten-free products. In the study of Singh and Whelan [44], the general availability of gluten-free products was limited, but there were differences in the availability between various types of stores, as the greatest availability characterized regular supermarkets, while budget supermarkets and corner shops barely had gluten-free versions of products. It must be mentioned that a British study conducted in 2024 demonstrated that from the coeliac disease patient’s perspective, the availability of gluten-free products in supermarkets, cafes, and restaurants has improved over time, but the availability in smaller and local shops still remains a struggle for those patients [45].

One of the findings derived from the present study is that younger respondents more often declared problems with the availability of some gluten-free alternatives to gluten-containing products, such as instant soups and beer, compared to older ones. Therefore, it may be suggested that the necessity of following a strict gluten-free diet and the resulting problems with the availability of gluten-free products may affect patients’ diets and their food choices in two different ways. On the one hand, it may result in improper nutritional quality of a diet [46] and lower intake of carbohydrates in coeliac disease patients [47], but on the other hand, based on the study of Kautto et al. [48], it may lead to reduced intake of some foods not recommended in a properly-balanced diet, as indicated for comfort foods, such as pizza and pastries.

As was noticed in the present study, a problem with the availability of gluten-free products may depend on the place of residence. It turned out that patients diagnosed with coeliac disease or their caregivers living in small towns may find it more difficult to buy specific gluten-free products, such as powder sauces, or non-food products, such as body and face cosmetics, and hair care cosmetics, compared to those living in big cities. Other research also suggests rural and urban areas differ in the variety and availability of gluten-free products [49], as in the study of Jamieson et al. [50], it was found that the average number of gluten-free products per shop in urban areas in Canada was four times greater than in rural areas. Consequently, the low availability of gluten-free products may translate into poor adherence to the gluten-free diet [51], which was observed in the study of Posterick and Ayars [52], in which rural residents with coeliac disease displayed significantly worse dietary adherence to gluten-free diet compared to residents of urban areas. Therefore, it is suggested that resources and efforts should be directed to nonurban communities to improve adherence to a gluten-free diet and positively influence patients’ well-being [52].

The problem with availability of gluten-free products is among other challenges and barriers which make a strict gluten-free diet hard to follow. First of all, coeliac patients often do not have an adequate level of knowledge to choose food products that do not pose any risk of gluten exposure [53]. Moreover, a gluten-free diet may constitute an economic burden for coeliac disease patients, as in the American study of Lee et al. [54], it was found that gluten-free products were 183% more expensive than their gluten-containing counterparts. Another problem encountered while following a gluten-free diet is the limited availability of gluten-free foods not only in markets, but also in restaurants [55]. Last but not least, following a gluten-free diet may affect coeliac patients’ social life, contributing to social isolation, as they may not participate in regular activities which are associated with the consumption of gluten-containing products [13].

The other problem is associated with the quality of gluten-free products. A recent review by Wieser et al. [16], assessing the frequency of exceeding the gluten content limits in a products labeled as gluten-free, naturally gluten-free products, or certified gluten-free products in various countries indicated that even more than 30% of assessed samples of gluten-free labeled products in United States, or India, as well as naturally gluten-free products in United States were gluten-contaminated with its content exceeding the limit of 20 ppm of gluten [12]. The other problem is general lower nutrition value of the gluten-free products than for their gluten-containing counterparts, including lower content of protein, iron, folic acid, thiamin, riboflavin, niacin, and fiber [14]. As a result, the risk of development of the non-communicable diseases, as a consequence of following not properly balanced diet, is indicated as a critical problem for coeliac disease patients, which should be managed by improving the nutritional quality of a gluten-free products [56]. It must be highlighted that improperly balanced gluten-free diet may increase the risk of obesity, metabolic syndrome, as well as may negatively influence lipid and glucose metabolism [10]. In United States the follow-up data from the Nurses’ Health Study (NHS), NHS II and Health Professionals Follow-Up Study (HPFS) indicated that such negative impact of gluten-free products influences inverse association between gluten intake and type 2 diabetes risk [57]. Another possible metabolic consequence of inadequately balanced gluten-free diet is a higher risk of non-alcoholic fatty liver disease among coeliac patients [58].

The conducted study confirms that there is a problem with the availability of non-cereal gluten-free products, which was declared by coeliac patients for gluten-free alternatives of non-cereal products, and it may be stated that it may result in lower adherence to a gluten-free diet, and potential gluten cross-contamination exposure risk. At the same time, education should be indicated as a potential action [59], that should be taken to provide adequate information for coeliac disease patients and to inform them about the potential risk of gluten exposure [60]. However, within such an education, dietary advice in terms of limiting the intake of sugar and saturated fatty acids to prevent the long-term metabolic consequences of a following a gluten-free diet, should be included [58].

### Limitations and Future Research

In spite of the fact that the present study provided some important data, it also has several limitations. Firstly, as a cross-sectional study based on the shopping behavior description, it does not establish causality between shopping behaviour and risk of gluten exposure [61], as no assessment of gluten content was conducted. What is more, as a cross-sectional study it does not allow assessing any changes in purchasing habits over time. However, it must be pointed out that the longitudinal studies concerning the availability of gluten-free products or consumer behaviors of coeliac patients are scarce, with predominant number of cross-sectional studies [28,55]. Longitudinal study, though, would allow to observe some trends or changes in purchasing habits among coeliac patients, like in the study of Vriesekoop et al. [62], which assessed changes in the availability and prices of gluten-free products between 2015 and 2019.

Another limitation associated with the present study is that it focused exclusively on Polish female coeliac patients and their caregivers, not including other nationalities and male patients. Therefore, the representativeness of the gathered sample cannot be concluded neither for international population, nor for Polish [63], as for Poland there were so far no studies describing the detailed characteristics of the coeliac disease patients and their families, so there are no data to be referred to assess if the characteristics of the group may allow to conclude it [64]. However, it is shown that purchasing habits may significantly vary depending on the place of residence [65] and cultural differences [66], so conducting the present study in a broader sample comprising both Polish and non-Polish residents may have influenced the obtained results due to the different range of gluten-free products in different countries.

The other limitation is associated with the fact that within the study the risk of potential gluten exposure, due to non-cereal products’ gluten cross-contamination risk was assessed only based on the fact that respondents declared choice of products not labeled as gluten-free, while the assessment of the quality of gluten-free products available on the Polish market was not conducted. While assessing the risk of potential gluten exposure, it should be taken into account, that the gluten content limits (20 ppm of gluten) is sometimes exceeded in case of products labeled as gluten-free, and even those certified, as well as that it is sometimes not exceeded in case of products naturally gluten-free, but not labelled [16]. Taking this into account, it should be emphasized that the study presents only the assessment of choice of products focusing on the potential higher or lower risk of gluten exposure, but without a real verification of gluten content.

What is more, the presented study, similarly to the other studies based on self-reporting, may be burdened with the related risk of bias [67]. In the group of coeliac patients, a problem associated with self-reporting bias may be quite common, as in the study of Leffler et al. [42] it was found that the level of tissue transglutaminase was elevated in the patients self-reporting a strict adherence to a gluten-free diet, which meant they actually may have been not highly adherent. Similarly, Smeets et al. [68] acknowledged in their study on coeliac patients that self-reporting may lead to data inaccuracies.

Within the present study, the frequency of not all non-cereal gluten free products was assessed, as only the most commonly consumed ones were taken into account. This may be treated as another limitation of the present study. The listed products were based on the questionnaire of de Koning et al. [28], including both cereal and non-cereal gluten-free products.

Last but not least, some possibly important factors were not studied, such as socio-economic status [69] or participants’ physical activity [70], while they might have influenced the obtained results.

To minimize the influence of the limitations mentioned above, future research should include longitudinal approaches to assess changes over time [71]. Moreover, to increase the possibility of the generalizability of the obtained results, the research planned ahead should include an international study sample comprising not only Polish female coeliac patients but also other nationalities and male patients. Such an approach will enable data extrapolation to a broader population of coeliac patients and will indicate the possible risk of gluten-cross contamination more adequately. In order to reduce the self-reporting bias, the biomarkers to monitor the adherence to a diet should be included [72], as the declaration of respondent may lead to subjective and inaccurate assessment of the level of adherence to gluten-free diet, including the lack of reporting the intentional or unintentional consumption of gluten. Furthermore, the study should not only focus on the choice of products (labeled as gluten-free or not), but it should also include the assessment of the quality of gluten-free products available on the Polish market. Another important issue to be addressed in future research is the assessment of nutritional value of gluten-free products and inclusion of broader selection of non-cereal gluten-free products in the analysis.

## 5. Conclusions

The majority of patients diagnosed with coeliac disease do not buy a number of gluten-free alternatives of the ‘hidden’ gluten sources, which means that they may be prone to gluten exposure, due to non-cereal products’ gluten cross-contamination risk. In general, respondents who most often purchased gluten-free alternatives of non-cereal products more often than other respondents, declared problems with their availability, which was stated in case of caregivers of coeliac disease patients (while compared with respondents diagnosed with coeliac disease), and older respondents (while compared with younger). The presented study provided insight into the needs of coeliac patients, declaring problems with the availability of gluten-free products. Taking this into account, the food producers should be aware, that there is a demand for a non-cereal products certified or labeled gluten-free, but also the education of patients diagnosed with coeliac disease should include issues associated with non-cereal products gluten cross-contamination exposure risk. Moreover, the further studies should compare the situation observed in Poland with the situation in the other countries.

## Figures and Tables

**Table 1:** Characteristics of the studied Polish female population of patients diagnosed with coeliac disease and their caregivers declaring at least occasionally purchasing gluten-free products online (*n* = 699).

Variable	Categories	Number of Respondents (%)
Age (years)	18–24	44 (6.29%)
	25–34	216 (30.90%)
	35–44	318 (45.49%)
	45–54	104 (14.88%)
	55–64	16 (2.29%)
	65–74	1 (0.14%)
Place of residence	Big city	327 (46.78%)
	Small town/village	372 (53.22%)
Major grocery shopping	Hypermarkets	494 (70.67%)
	Other	205 (29.33%)
Coeliac disease	Respondent diagnosed	445 (63.66%)
	Family member/relative diagnosed	254 (36.34%)
Problem with gluten-free products availability	Not declared	19 (2.72%)
	Declared	680 (97.28%)
Problem with gluten-free products quality	Not declared	268 (38.34%)
	Declared	431 (61.66%)

**Table 2:** The frequency of buying gluten-free alternatives of the ‘hidden’ gluten sources declared in the studied Polish female population of patients diagnosed with coeliac disease and their caregivers declaring at least occasionally purchasing gluten-free products online (*n* = 699).

Food Product Groups (Number of Respondents)	Never	Rarely	Sometimes	Often
Baking powder (*n* = 698)	43 (6.2%)	92 (13.2%)	243 (34.8%)	320 (45.8%)
Snack bars (*n* = 692)	80 (11.6%)	225 (32.5%)	259 (37.4%)	128 (18.5%)
Powder sauces (*n* = 696)	553 (79.5%)	106 (15.2%)	26 (3.7%)	11 (1.6%)
Soups (*n* = 694)	506 (72.9%)	133 (19.2%)	44 (6.3%)	11 (1.6%)
Stock cubes (*n* = 694)	218 (31.4%)	245 (35.3%)	154 (22.2%)	77 (11.1%)
Side dishes (*n* = 698)	45 (6.4%)	117 (16.8%)	270 (38.7%)	266 (38.1%)
Spices (*n* = 696)	95 (13.6%)	99 (14.2%)	179 (25.7%)	323 (46.4%)
Instant soups (*n* = 696)	584 (83.9%)	89 (12.8%)	18 (2.6%)	5 (0.7%)
Chocolate and chocolate products (*n* = 693)	39 (5.6%)	158 (22.8%)	323 (46.6%)	173 (25.0%)
Candies (*n* = 698)	109 (15.6%)	221 (31.7%)	246 (35.2%)	122 (17.5%)
Ice cream (*n* = 697)	33 (4.7%)	146 (20.9%)	287 (41.2%)	231 (33.1%)
Beer (*n* = 688)	386 (56.1%)	163 (23.7%)	98 (14.2%)	41 (6.0%)
Body and face cosmetics (*n* = 689)	249 (36.1%)	134 (19.4%)	153 (22.2%)	153 (22.2%)
Hair care cosmetics (*n* = 688)	265 (38.5%)	129 (18.8%)	148 (21.5%)	146 (21.2%)

**Table 3:** The problems with the availability of gluten-free alternatives of the ‘hidden’ gluten sources declared in the studied Polish female population of patients diagnosed with coeliac disease and their caregivers declaring at least occasionally purchasing gluten-free products online (*n* = 699).

Food Product Groups (Number of Respondents)	Declared Problems	No Problems Declared
Baking powder (*n* = 656)	192 (29.3%)	464 (70.7%)
Snack bars (*n* = 619)	74 (12.0%)	545 (88.0%)
Powder sauces (*n* = 146)	38 (26.0%)	108 (74.0%)
Soups (*n* = 193)	70 (36.3%)	123 (63.7%)
Stock cubes (*n* = 481)	53 (11.0%)	428 (89.0%)
Side dishes (*n* = 654)	56 (8.6%)	598 (91.4%)
Spices (*n* = 604)	194 (32.1%)	410 (67.9%)
Instant soups (*n* = 115)	42 (36.5%)	73 (63.5%)
Chocolate and chocolate products (*n* = 660)	91 (13.8%)	569 (86.2%)
Candies (*n* = 590)	51 (8.6%)	539 (91.4%)
Ice cream (*n* = 666)	89 (13.4%)	577 (86.6%)
Beer (*n* = 313)	136 (43.5%)	177 (56.5%)
Body and face cosmetics (*n* = 450)	27 (6.0%)	423 (94.0%)
Hair care cosmetics (*n* = 434)	20 (4.6%)	414 (95.4%)

**Table 4:** The frequency of buying gluten-free alternatives of the ‘hidden’ gluten sources declared in the studied Polish female population of patients diagnosed with coeliac disease and their caregivers declaring at least occasionally purchasing gluten-free products online (*n* = 699), stratified by diagnosis of coeliac disease.

Food Product Groups (Number of Respondents)	Diagnosed with Coeliac Disease			Caregiver of a Patient with Coeliac Disease			Cramer’s V	*p*-Value
	Never	Rarely/Sometimes	Often	Never	Rarely/Sometimes	Often		
Baking powder (*n* = 445/253)	36 (8.1%)	226 (50.8%)	183 (41.1%)	7 (2.8%)	109 (43.1%)	137 (54.2%)	0.1716	0.0005
Snack bars (*n* = 441/251)	62 (14.1%)	306 (69.4%)	73 (16.6%)	18 (7.2%)	178 (70.9%)	55 (21.9%)	0.1197	0.0106
Powder sauces (*n* = 443/253)	358 (80.8%)	81 (18.3%)	4 (0.9%)	195 (77.1%)	51 (20.2%)	7 (2.8%)	0.0846	0.1274
Soups (*n* = 440/254)	331 (75.2%)	104 (23.6%)	5 (1.1%)	175 (68.9%)	73 (28.7%)	6 (2.4%)	0.0781	0.1316
Stock cubes (*n* = 440/254)	142 (32.3%)	249 (56.6%)	49 (11.1%)	76 (29.9%)	150 (59.1%)	28 (11.0%)	0.0406	0.7964
Side dishes (*n* = 445/253)	32 (7.2%)	247 (55.5%)	166 (37.3%)	13 (5.1%)	140 (55.3%)	100 (39.5%)	0.0441	0.5315
Spices (*n* = 443/253)	69 (15.6%)	176 (39.7%)	198 (44.7%)	26 (10.3%)	102 (40.3%)	125 (49.4%)	0.0878	0.1289
Instant soups (*n* = 443/253)	383 (86.5%)	57 (12.9%)	3 (0.7%)	201 (79.4%)	50 (19.8%)	2 (0.8%)	0.0929	0.0510
Chocolate and chocolate products (*n* = 440/253)	32 (7.3%)	303 (68.9%)	105 (23.9%)	7 (2.8%)	178 (70.4%)	68 (26.0%)	0.1527	0.0401
Candies (*n* = 444/254)	91 (20.5%)	286 (64.4%)	67 (15.1%)	18 (7.1%)	181 (71.3%)	55 (21.7%)	0.2061	<0.0001
Ice cream (*n* = 443/254)	27 (6.1%)	291 (65.7%)	125 (28.2%)	6 (2.4%)	142 (55.9%)	106 (41.7%)	0.1794	0.0003
Beer (*n* = 438/250)	209 (47.7%)	195 (44.5%)	34 (7.8%)	177 (70.8%)	66 (26.4%)	7 (2.8%)	0.2372	<0.0001
Body and face cosmetics (*n* = 438/251)	148 (33.8%)	181 (41.3%)	109 (24.9%)	101 (40.2%)	106 (42.2%)	44 (17.5%)	0.0943	0.0563
Hair care cosmetics (*n* = 437/251)	156 (35.7%)	178 (40.7%)	103 (23.6%)	109 (43.4%)	99 (39.4%)	43 (17.1%)	0.0948	0.0592

**Table 5:** The frequency of buying gluten-free alternatives of the ‘hidden’ gluten sources declared in the studied Polish female population of patients diagnosed with coeliac disease and their caregivers declaring at least occasionally purchasing gluten-free products online (*n* = 699), stratified by age.

Food Product Groups (Number of Respondents)	Age < 35 years			Age ≥ 35 years			Cramer’s V	*p*-Value
	Never	Rarely/Sometimes	Often	Never	Rarely/Sometimes	Often		
Baking powder (*n* = 260/438)	18 (6.9%)	139 (53.5%)	103 (39.6%)	25 (5.7%)	196 (44.7%)	217 (49.5%)	0.1221	0.0392
Snack bars (*n* = 258/434)	35 (13.6%)	176 (68.2%)	47 (18.2%)	45 (10.4%)	308 (71.0%)	81 (18.7%)	0.0796	0.4441
Powder sauces (*n* = 260/436)	209 (80.4%)	48 (18.5%)	3 (1.2%)	344 (78.9%)	84 (19.3%)	8 (1.8%)	0.0392	0.7487
Soups (*n* = 258/436)	196 (76.0%)	58 (22.5%)	4 (1.6%)	310 (71.1%)	119 (27.3%)	7 (1.6%)	0.0579	0.3675
Stock cubes (*n* = 259/435)	83 (32.0%)	147 (56.8%)	29 (11.2%)	135 (31.0%)	252 (57.9%)	48 (11.0%)	0.0598	0.9456
Side dishes (*n* = 260/438)	16 (6.2%)	146 (56.2%)	98 (37.7%)	29 (6.6%)	241 (55.0%)	168 (38.4%)	0.0300	0.9457
Spices (*n* = 259/437)	38 (14.7%)	107 (41.3%)	114 (44.0%)	57 (13.0%)	171 (39.1%)	209 (47.8%)	0.0489	0.6009
Instant soups (*n* = 260/436)	221 (85.0%)	35 (13.5%)	4 (1.5%)	363 (83.3%)	72 (16.5%)	1 (0.2%)	0.0842	0.0850
Chocolate and chocolate products (*n* = 260/433)	18 (6.9%)	165 (63.5%)	77 (29.6%)	21 (4.8%)	316 (73.0%)	96 (22.0%)	0.1007	0.0307
Candies (*n* = 259/439)	42 (16.2%)	172 (66.4%)	45 (17.4%)	67 (15.3%)	295 (67.2%)	77 (17.5%)	0.0638	0.9452
Ice cream (*n* = 259/438)	13 (5.0%)	166 (64.1%)	80 (30.9%)	20 (4.6%)	267 (61.0%)	151 (34.5%)	0.0490	0.6189
Beer (*n* = 257/431)	136 (52.9%)	100 (38.9%)	21 (8.2%)	250 (58.0%)	161 (37.4%)	20 (4.6%)	0.1084	0.1217
Body and face cosmetics (*n* = 257/432)	88 (34.2%)	98 (38.1%)	71 (27.6%)	161 (37.3%)	189 (43.8%)	82 (19.0%)	0.1086	0.0294
Hair care cosmetics (*n* = 431/257)	93 (36.2%)	99 (38.5%)	65 (25.3%)	172 (39.9%)	178 (41.3%)	81 (18.8%)	0.0792	0.1293

**Table 6:** The frequency of buying gluten-free alternatives of the ‘hidden’ gluten sources declared in the studied Polish female population of patients diagnosed with coeliac disease and their caregivers declaring at least occasionally purchasing gluten-free products online (*n* = 699), stratified by place of residence.

Food Product Groups (Number of Respondents)	Living in a Big City			Living in a Small Town/Village			Cramer’s V	*p*-Value
	Never	Rarely/Sometimes	Often	Never	Rarely/Sometimes	Often		
Baking powder (*n* = 327/371)	23 (7.0%)	149 (45.6%)	155 (47.4%)	20 (5.4%)	186 (50.1%)	165 (44.5%)	0.0581	0.3981
Snack bars (*n* = 324/368)	34 (10.5%)	225 (69.4%)	65 (20.1%)	46 (12.5%)	259 (70.4%)	63 (17.1%)	0.0456	0.4897
Powder sauces (*n* = 326/370)	262 (80.4%)	59 (18.1%)	5 (1.5%)	291 (78.6%)	73 (19.7%)	6 (1.6%)	0.0485	0.8538
Soups (*n* = 323/371)	234 (72.4%)	82 (25.4%)	7 (2.2%)	272 (73.3%)	95 (25.6%)	4 (1.1%)	0.0438	0.5186
Stock cubes (*n* = 324/370)	103 (31.8%)	188 (58.0%)	33 (10.2%)	115 (31.1%)	211 (57.0%)	44 (11.9%)	0.0290	0.7745
Side dishes (*n* = 326/372)	21 (6.4%)	185 (56.7%)	120 (36.8%)	24 (6.5%)	202 (54.3%)	146 (39.2%)	0.0355	0.7951
Spices (*n* = 326/370)	49 (15.0%)	130 (39.9%)	147 (45.1%)	46 (12.4%)	148 (40.0%)	176 (47.6%)	0.0396	0.5808
Instant soups (*n* = 327/369)	285 (87.2%)	40 (12.2%)	2 (0.6%)	299 (81.0%)	67 (18.2%)	3 (0.8%)	0.0900	0.0893
Chocolate and chocolate products (*n* = 323/370)	20 (6.2%)	219 (67.8%)	84 (26.0%)	19 (5.1%)	262 (70.8%)	89 (24.1%)	0.0458	0.6602
Candies (*n* = 327/371)	56 (17.1%)	215 (65.7%)	56 (17.1%)	53 (14.3%)	252 (67.9%)	66 (17.8%)	0.0514	0.5873
Ice cream (*n* = 327/370)	15 (4.6%)	190 (58.1%)	122 (37.3%)	18 (4.9%)	243 (65.7%)	109 (29.5%)	0.0848	0.0882
Beer (*n* = 324/364)	186 (57.4%)	117 (36.1%)	21 (6.5%)	200 (54.9%)	144 (39.6%)	20 (5.5%)	0.0789	0.6056
Body and face cosmetics (*n* = 322/367)	126 (39.1%)	130 (40.4%)	66 (20.5%)	123 (33.5%)	157 (42.8%)	87 (23.7%)	0.0617	0.2822
Hair care cosmetics (*n* = 322/366)	133 (41.3%)	128 (39.8%)	61 (18.9%)	132 (36.1%)	149 (40.7%)	85 (23.2%)	0.0632	0.2543

**Table 7:** The frequency of buying gluten-free alternatives of the ‘hidden’ gluten sources declared in the studied Polish female population of patients diagnosed with coeliac disease and their caregivers declaring at least occasionally purchasing gluten-free products online (*n* = 699), stratified by primary place of purchasing major grocery shopping.

Food Product Groups (Number of Respondents)	Purchasing in Hypermarkets			Purchasing in Shops Other Than Hypermarkets			Cramer’s V	*p*-Value
	Never	Rarely/Sometimes	Often	Never	Rarely/Sometimes	Often		
Baking powder (*n* = 205/493)	24 (11.7%)	96 (46.8%)	85 (41.5%)	19 (3.9%)	239 (48.5%)	235 (47.7%)	0.1693	0.0004
Snack bars (*n* = 203/489)	25 (12.3%)	151 (74.4%)	27 (13.3%)	55 (11.2%)	333 (68.1%)	101 (20.7%)	0.0910	0.0763
Powder sauces (*n* = 204/492)	161 (78.9%)	40 (19.6%)	3 (1.5%)	392 (79.7%)	92 (19.6%)	8 (1.6%)	0.0119	0.9536
Soups (*n* = 204/490)	158 (77.5%)	44 (21.6%)	2 (1.0%)	348 (71.0%)	133 (27.1%)	9 (1.8%)	0.0805	0.1981
Stock cubes (*n* = 203/491)	68 (33.5%)	116 (57.1%)	19 (9.4%)	150 (30.4%)	283 (57.6%)	58 (11.8%)	0.0550	0.5537
Side dishes (*n* = 205/493)	13 (6.3%)	122 (59.5%)	70 (34.1%)	32 (6.5%)	265 (53.8%)	196 (39.8%)	0.0605	0.3557
Spices (*n* = 205/491)	20 (9.8%)	95 (46.3%)	90 (43.9%)	75 (15.3%)	183 (37.3%)	233 (47.5%)	0.1066	0.0369
Instant soups (*n* = 204/492)	174 (85.3%)	29 (14.2%)	1 (0.5%)	410 (83.3%)	78 (15.9%)	4 (0.8%)	0.0489	0.7684
Chocolate and chocolate products (*n* = 201/492)	9 (4.5%)	147 (73.1%)	45 (22.4%)	30 (6.1%)	334 (67.9%)	128 (26.0%)	0.0553	0.3708
Candies (*n* = 204/494)	33 (16.2%)	147 (72.1%)	24 (11.8%)	76 (15.4%)	320 (64.8%)	98 (19.8%)	0.1030	0.0371
Ice cream (*n* = 205/492)	8 (3.9%)	140 (68.3%)	57 (27.8%)	25 (5.1%)	293 (59.6%)	174 (35.4%)	0.0824	0.0954
Beer (*n* = 201/487)	115 (57.2%)	77 (38.3%)	9 (4.5%)	271 (55.6%)	184 (37.8%)	32 (6.6%)	0.0751	0.5716
Body and face cosmetics (*n* = 204/485)	65 (31.9%)	95 (46.6%)	44 (21.6%)	184 (37.9%)	192 (39.6%)	109 (22.5%)	0.0730	0.2019
Hair care cosmetics (*n* = 204/484)	70 (34.3%)	91 (44.6%)	43 (21.1%)	195 (40.3%)	186 (38.4%)	103 (21.3%)	0.0647	0.2601

**Table 8:** The problems with the availability of gluten-free alternatives of the ‘hidden’ gluten sources declared in the studied Polish female population of patients diagnosed with coeliac disease and their caregivers declaring at least occasionally purchasing gluten-free products online (*n* = 699), stratified by diagnosis of coeliac disease.

Food Product Groups (Number of Respondents)	Diagnosed with Coeliac Disease		Caregiver of a Patient with Coeliac Disease		Phi (φ)	*p*-Value
	Declared Problems	No Problems Declared	Declared Problems	No Problems Declared		
Baking powder (*n* = 409/246)	129 (29.0%)	316 (71.0%)	84 (33.1%)	170 (66.9%)	−0.0426	0.2973
Snack bars (*n* = 409/246)	54 (12.1%)	391 (87.9%)	31 (12.2%)	223 (87.8%)	−0.0010	0.9258
Powder sauces (*n* = 409/246)	75 (16.9%)	370 (83.1%)	48 (18.9%)	206 (81.1%)	−0.0258	0.5624
Soups (*n* = 409/246)	106 (23.8%)	339 (76.2%)	73 (28.7%)	181 (71.3%)	−0.0542	0.1792
Stock cubes (*n* = 409/246)	44 (9.9%)	401 (90.1%)	31 (12.2%)	223 (87.8%)	−0.0360	0.4094
Side dishes (*n* = 409/246)	37 (8.3%)	408 (91.7%)	26 (10.2%)	228 (89.8%)	−0.0323	0.4740
Spices (*n* = 409/246)	127 (28.5%)	318 (71.5%)	110 (43.3%)	144 (56.7%)	−0.1500	<0.0001
Instant soups (*n* = 409/246)	84 (18.9%)	361 (81.1%)	51 (20.1%)	203 (79.9%)	−0.1465	0.7736
Chocolate and chocolate products (*n* = 409/246)	53 (11.9%)	392 (88.1%)	46 (18.1%)	208 (81.9%)	−0.0855	0.0317
Candies (*n* = 409/246)	40 (9.0%)	405 (91.0%)	20 (7.9%)	234 (92.1%)	−0.0191	0.7146
Ice cream (*n* = 409/246)	58 (13.0%)	387 (87.0%)	37 (14.6%)	217 (85.4%)	−0.0215	0.6497
Beer (*n* = 409/246)	151 (33.9%)	294 (66.1%)	41 (16.1%)	213 (83.9%)	−0.1917	<0.0001
Body and face cosmetics (*n* = 409/246)	28 (6.3%)	417 (93.7%)	19 (7.5%)	235 (92.5%)	−0.0228	0.6554
Hair care cosmetics (*n* = 409/246)	24 (5.4%)	421 (94.6%)	16 (6.3%)	238 (93.7%)	−0.0188	0.7439

**Table 9:** The problems with the availability of gluten-free alternatives of the ‘hidden’ gluten sources declared in the studied Polish female population of patients diagnosed with coeliac disease and their caregivers declaring at least occasionally purchasing gluten-free products online (*n* = 699), stratified by age.

Food Product Groups (Number of Respondents)	Age < 35 Years		Age ≥ 35 Years		Phi (φ)	*p*-Value
	Declared Problems	No Problems Declared	Declared Problems	No Problems Declared		
Baking powder (*n* = 260/439)	82 (31.5%)	178 (68.5%)	131 (29.8%)	308 (70.2%)	0.0178	0.6992
Snack bars (*n* = 260/439)	36 (13.8%)	224 (86.2%)	49 (11.2%)	390 (88.8%)	0.0397	0.3525
Powder sauces (*n* = 260/439)	54 (20.8%)	206 (79.2%)	69 (15.7%)	370 (84.3%)	0.0641	0.1113
Soups (*n* = 260/439)	75 (28.8%)	185 (71.2%)	104 (23.7%)	335 (76.3%)	0.0571	0.1557
Stock cubes (*n* = 260/439)	35 (13.5%)	225 (86.5%)	40 (9.1%)	399 (90.9%)	0.0679	0.0950
Side dishes (*n* = 260/439)	29 (11.2%)	231 (88.8%)	34 (7.7%)	405 (92.3%)	0.0575	0.1662
Spices (*n* = 260/439)	96 (36.9%)	164 (63.1%)	141 (32.1%)	298 (67.9%)	0.0491	0.2246
Instant soups (*n* = 260/439)	61 (23.5%)	199 (76.5%)	74 (16.9%)	365 (83.1%)	0.0809	0.0415
Chocolate and chocolate products (*n* = 260/439)	32 (12.3%)	228 (87.7%)	67 (15.3%)	372 (84.7%)	−0.0410	0.3318
Candies (*n* = 260/439)	26 (10.0%)	234 (90.0%)	34 (7.7%)	405 (92.3%)	−0.0389	0.3740
Ice cream (*n* = 260/439)	37 (14.2%)	223 (85.8%)	58 (13.2%)	381 (86.8%)	−0.0144	0.7904
Beer (*n* = 260/439)	90 (34.6%)	170 (65.4%)	102 (23.2%)	337 (76.8%)	−0.1232	0.0015
Body and face cosmetics (*n* = 260/439)	12 (4.6%)	248 (95.4%)	35 (8.0%)	404 (92.0%)	−0.0648	0.1195
Hair care cosmetics (*n* = 260/439)	11 (4.2%)	249 (95.8%)	29 (6.6%)	410 (93.4%)	−0.0494	0.2550

**Table 10:** The problems with the availability of gluten-free alternatives of the ‘hidden’ gluten sources declared in the studied Polish female population of patients diagnosed with coeliac disease and their caregivers declaring at least occasionally purchasing gluten-free products online (*n* = 699), stratified by place of residence.

Food Product Groups (Number of Respondents)	Living in a Big City		Living in a Small Town/Village		Phi (φ)	*p*-Value
	Declared Problems	No Problems Declared	Declared Problems	No Problems Declared		
Baking powder (*n* = 327/372)	103 (31.5%)	224 (68.5%)	110 (29.6%)	262 (70.4%)	0.0209	0.6381
Snack bars (*n* = 327/372)	43 (13.1%)	284 (86.9%)	42 (11.3%)	330 (88.7%)	0.0284	0.5257
Powder sauces (*n* = 327/372)	44 (13.5%)	283 (86.5%)	79 (21.2%)	293 (78.8%)	−0.1020	0.0094
Soups (*n* = 327/372)	79 (24.2%)	248 (75.8%)	100 (26.9%)	272 (73.1%)	−0.0311	0.4617
Stock cubes (*n* = 327/372)	38 (11.6%)	289 (88.4%)	37 (9.9%)	335 (90.1%)	0.0270	0.5543
Side dishes (*n* = 327/372)	31 (9.5%)	296 (90.5%)	32 (8.6%)	340 (91.4%)	0.0153	0.7856
Spices (*n* = 327/372)	109 (33.3%)	218 (66.7%)	128 (34.4%)	244 (65.6%)	−0.0113	0.8262
Instant soups (*n* = 327/372)	57 (17.4%)	270 (82.6%)	78 (21.0%)	294 (79.0%)	−0.0447	0.2776
Chocolate and chocolate products (*n* = 327/372)	48 (14.7%)	279 (85.3%)	51 (13.7%)	321 (86.3%)	0.0139	0.7964
Candies (*n* = 327/372)	29 (8.9%)	298 (91.1%)	31 (8.3%)	341 (91.7%)	0.0095	0.9071
Ice cream (*n* = 327/372)	48 (14.7%)	279 (85.3%)	47 (12.6%)	325 (87.4%)	0.0298	0.4988
Beer (*n* = 327/372)	96 (29.4%)	231 (70.6%)	96 (25.8%)	276 (74.2%)	0.0397	0.3347
Body and face cosmetics (*n* = 327/372)	13 (4.0%)	314 (96.0%)	34 (9.1%)	338 (90.9%)	−0.1029	0.0102
Hair care cosmetics (*n* = 327/372)	11 (3.4%)	316 (96.6%)	29 (7.8%)	343 (92.2%)	−0.0952	0.0186

**Table 11:** The problems with the availability of gluten-free alternatives of the ‘hidden’ gluten sources declared in the studied Polish female population of patients diagnosed with coeliac disease and their caregivers declaring at least occasionally purchasing gluten-free products online (*n* = 699), stratified by primary place of purchasing major grocery shopping.

Food Product Groups (Number of Respondents)	Purchasing in Hypermarkets		Purchasing in Shops Other Than Hypermarkets		Phi (φ)	*p*-Value
	Declared Problems	No Problems Declared	Declared Problems	No Problems Declared		
Baking powder (*n* = 494/205)	142 (28.7%)	352 (71.3%)	71 (34.6%)	134 (65.4%)	−0.0582	0.1471
Snack bars (*n* = 494/205)	63 (12.8%)	431 (87.2%)	22 (10.7%)	183 (89.3%)	0.0282	0.5370
Powder sauces (*n* = 494/205)	93 (18.8%)	401 (81.2%)	30 (14.6%)	175 (85.4%)	0.0501	0.2240
Soups (*n* = 494/205)	131 (26.5%)	363 (73.5%)	48 (23.4%)	157 (76.6%)	0.0324	0.4468
Stock cubes (*n* = 494/205)	51 (10.3%)	443 (89.7%)	24 (11.7%)	181 (88.3%)	−0.0204	0.6864
Side dishes (*n* = 494/205)	51 (10.3%)	443 (89.7%)	12 (5.9%)	193 (94.1%)	0.0711	0.0829
Spices (*n* = 494/205)	170 (34.4%)	324 (65.6%)	67 (32.7%)	138 (67.3%)	0.0166	0.7247
Instant soups (*n* = 494/205)	104 (21.1%)	390 (78.9%)	31 (15.1%)	174 (84.9%)	0.0682	0.0886
Chocolate and chocolate products (*n* = 494/205)	71 (14.4%)	423 (85.6%)	28 (13.7%)	177 (86.3%)	0.0093	0.8987
Candies (*n* = 494/205)	43 (8.7%)	451 (91.3%)	17 (8.3%)	188 (91.7%)	0.0067	0.9772
Ice cream (*n* = 494/205)	72 (14.6%)	422 (85.4%)	23 (11.2%)	182 (88.8%)	0.0445	0.2904
Beer (*n* = 494/205)	139 (28.1%)	355 (71.9%)	53 (25.9%)	152 (74.1%)	0.0233	0.6011
Body and face cosmetics (*n* = 494/205)	34 (6.9%)	460 (93.1%)	13 (6.3%)	192 (93.7%)	0.0098	0.9249
Hair care cosmetics (*n* = 494/205)	30 (6.1%)	464 (93.9%)	10 (4.9%)	195 (95.1%)	0.0234	0.6597

**Table 12:** The problems with the availability of gluten-free alternatives of the ‘hidden’ gluten sources declared in the studied Polish female population of patients diagnosed with coeliac disease and their caregivers declaring at least occasionally purchasing gluten-free products online (*n* = 699), stratified by the frequency of buying them.

Food Product Groups (Number of Respondents)	Rarely		Sometimes		Often		Cramer’s V	*p*-Value
	Declared Problems	No Problems Declared	Declared Problems	No Problems Declared	Declared Problems	No Problems Declared		
Baking powder (*n* = 655)	87 (13.3%)	5 (0.8%)	236 (36.0%)	7 (1.1%)	314 (47.9%)	6 (0.9%)	0.0705	0.1814
Snack bars (*n* = 612)	217 (35.5%)	8 (1.3%)	252 (41.2%)	7 (1.1%)	128 (20.9%)	0 (0%)	0.0907	0.1090
Powder sauces (*n* = 143)	104 (72.7%)	2 (1.4%)	26 (18.2%)	0 (0%)	11 (7.7%)	0 (0%)	0.0475	0.7019
Soups (*n* = 188)	129 (68.6%)	4 (2.1%)	44 (23.4%)	0 (0%)	11 (5.9%)	0 (0%)	0.0492	0.4296
Stock cubes (*n* = 476)	237 (49.8%)	8 (1.7%)	153 (32.1%)	1 (0.2%)	77 (16.2%)	0 (0%)	0.1056	0.0721
Side dishes (*n* = 653)	106 (16.2%)	11 (1.7%)	266 (40.7%)	4 (0.6%)	263 (40.3%)	3 (0.5%)	0.1850	<0.0001
Spices (*n* = 601)	97 (16.1%)	2 (0.3%)	172 (28.6%)	7 (1.2%)	318 (53.0%)	5 (0.8%)	0.0858	0.2374
Instant soups (*n* = 112)	87 (77.6%)	2 (1.8%)	18 (16.1%)	0 (0%)	5 (4.5%)	0 (0%)	0.0338	0.7687
Chocolate and chocolate products (*n* = 654)	150 (22.9%)	8 (1.2%)	314 (48.0%)	9 (1.4%)	173 (26.5%)	0 (0%)	0.1134	0.0146
Candies (*n* = 589)	217 (36.8%)	4 (0.7%)	239 (40.6%)	7 (1.2%)	122 (20.7%)	0 (0%)	0.1359	0.1645
Ice cream (*n* = 664)	140 (21.1%)	6 (0.9%)	279 (42.0%)	8 (1.2%)	230 (34.6%)	1 (0.2%)	0.1544	0.0565
Beer (*n* = 302)	159 (52.7%)	4 (1.3%)	97 (32.1%)	1 (0.3%)	41 (13.6%)	0 (0%)	0.0703	0.4559
Body and face cosmetics (*n* = 440)	132 (30.0%)	2 (0.5%)	147 (33.4%)	6 (1.4%)	153 (34.7%)	0 (0%)	0.1109	0.0350
Hair care cosmetics (*n* = 423)	126 (29.8%)	3 (0.7%)	141 (33.3%)	7 (1.7%)	146 (34.5%)	0 (0%)	0.0992	0.0284

## Data Availability

Data provided on request.
